# Supplementary material for: FERN – a Java framework for stochastic simulation and evaluation of reaction networks
Source: BMC Bioinformatics. 2008 Aug 29;9:356. doi: 10.1186/1471-2105-9-356 (PMC2553347; doi:10.1186/1471-2105-9-356)
Supplement: Additional file 1 — FERN distribution, Version 1.3. This archive contains the FERN source code and binaries as well as documentation and example models in FernML and SBML. [file 1471-2105-9-356-S1.zip › fern/doc/javadoc/fern/tools/functions/package-use.html]

Uses of Package fern.tools.functions


---


|  |  |  |
| --- | --- | --- |
| **Overview** \| **Package** \| Class \| **Use** \| **Tree** \| **Deprecated** \| **Index** \| **Help** | |  |
| PREV   NEXT | **FRAMES**    **NO FRAMES**     **All Classes** |  |


---


## **Uses of Package fern.tools.functions**

| Packages that use fern.tools.functions | |
| --- | --- |
| **fern.network.creation** | Provides classes for the evolution of networks. |
| **fern.tools.functions** | Provides common function delegates and implementations. |

Classes in fern.tools.functions used by fern.network.creation

****Probability****
  
          Function delegate for conditional probabilities.

Classes in fern.tools.functions used by fern.tools.functions

****Probability****
  
          Function delegate for conditional probabilities.

---


|  |  |  |
| --- | --- | --- |
| **Overview** \| **Package** \| Class \| **Use** \| **Tree** \| **Deprecated** \| **Index** \| **Help** | |  |
| PREV   NEXT | **FRAMES**    **NO FRAMES**     **All Classes** |  |


---
